# Early diagnosis of Crohn’s disease in patients presenting with a perianal fistula: systematic review and development of a perianal red flags index

**DOI:** 10.1007/s10151-024-03106-y

**Published:** 2025-03-28

**Authors:** L. J. Munster, G. R. Meriba, J. Schuitema, S. van Dieren, E. J. de Groof, M. W. Mundt, G. R. D’Haens, W. A. Bemelman, C. J. Buskens, J. D. W. van der Bilt

**Affiliations:** 1 Department of Surgery, Flevoziekenhuis, Almere, The Netherlands; 2 Department of Surgery, Amsterdam UMC (Location VUmc), De Boelelaan 1117, 1081 HV Amsterdam, The Netherlands; 3 Department of Gastroenterology and Hepatology, Flevoziekenhuis, Almere, The Netherlands; 4 Department of Gastroenterology and Hepatology, Amsterdam UMC (Location VUmc), Amsterdam, The Netherlands

**Keywords:** Perianal fistula, Crohn’s disease, Inflammatory bowel disease, Delay

## Abstract

**Background:**

Delay in diagnosing Crohn’s disease (CD) in patients presenting with perianal abscess (PAA) and/or fistula (PAF) is common. The aim of this study was to identify red flags suggestive of CD.

**Methods:**

A systematic literature review was conducted to identify symptoms associated with CD in patients presenting with PAA/PAF. A questionnaire including those symptoms, supplemented with items from the International Organization for the Study of Inflammatory Bowel Diseases (IO-IBD) red flags index for luminal CD, was administered to all adult patients presenting with a PAF and eventually diagnosed with CD and matched patients (1:3) from the same study period with a cryptoglandular PAF (2012–2023) at a single non-academic teaching hospital. All patients were asked to recall symptoms/signs experienced during their first PAF.

**Results:**

The systematic review identified 8 articles reporting on 15 clinical characteristics in patients presenting with PAA (*n* = 2)/PAF (*n* = 6), supplemented with 13 items from the IO-IBD red flags index (28 items in total). A total of 25 patients with CD and 75 patients with PAF without CD answered the questionnaire. Univariate analysis identified seven items associated with CD (age, family history, > 2 perianal interventions, weight loss, abdominal pain, diarrhoea and fatigue), and four items remained significant in multivariate analysis: age (OR 3.4 [1.0–11.5]), > 2 previous perianal interventions (OR 3.4 [1.0–10.1]), weight loss (OR 14.4 [3.7–55.6]) and abdominal pain (OR 9.8 [1.9–49.8]). Receiver-operating characteristic curve (ROC) analysis showed that a combination of these red flags was associated with good discrimination of CD versus non-CD (AUC 0.83 [0.72–0.94]).

**Conclusions:**

The perianal red flags index has a good predictive value for early identification of patients with PAF at risk for underlying CD.

**Supplementary Information:**

The online version contains supplementary material available at 10.1007/s10151-024-03106-y.

## Introduction

Perianal abscesses (PAA) and perianal fistulas (PAF) are debilitating conditions associated with pain, discharge, restricted mobility and fatigue contributing to a significant reduction in quality of life (QoL) [1, 2]. The majority of PAA have a cryptoglandular origin and are caused by infection of the perianal crypt glands [2]. In about a third of patients with a PAA, a PAF remains as a tract connecting the intestinal lumen of the anal canal or rectum with the perianal skin and is found either at the time or subsequent to abscess drainage [3]. It was estimated that the overall incidence of PAF in Europe ranged from 1.2 to 2.8 per 10,000 people [4]. With surgical treatment strategies for cryptoglandular PAF, depending on the type of therapy, healing rates are approximately 70–80% [5–9].

The second most common cause of a PAF is Crohn’s disease (CD), often affecting young patients in the midst of their most active period in life [10]. In around one-third of all patients with CD-associated perianal disease, a PAA or PAF is a first manifestation of the disease and perianal surgery often precedes diagnosis of CD [1, 9, 11–15]. Due to the high incidence of idiopathic or cryptoglandular PAA/PAF in the general population, delay in diagnosis of CD in these patients is common as initial signs and symptoms can be non-specific. Time to CD diagnosis may take many years [16–36]. Differentiating Crohn’s from cryptoglandular PAF can be challenging, but is of the utmost importance, as CD-associated PAF are generally considered more refractory to surgery without IBD-directed medication [23, 24]. Treating these fistulas without proper multidisciplinary management will put patients at higher risk of unnecessary damage to the anal sphincter, progression of the disease and possible worse long-term outcomes and decreased quality of life (QoL).

The aim of this study was to identify signs and symptoms suggestive of CD in patients presenting with a PAA or PAF in a systematic literature review and a retrospective matched-controlled study to develop a perianal red flags instrument for early identification of patients at risk for underlying CD.

## Materials and methods

The study consisted of two parts. First, a systematic literature review of signs and symptoms suggestive of CD was conducted. Second, a retrospective matched cohort study was performed at a non-academic teaching centre in the Netherlands in all adult (≥ 16 year) patients presenting with a PAF and eventually diagnosed with CD between 2012 and 2023. Patients with CD were matched 1:3 with patients from the same study period with a cryptoglandular PAF.

### Part I: systematic review

#### Search strategy

A literature search was performed on the basis of the Preferred Reporting Items for Systematic Reviews and Meta-Analysis (PRISMA)-statement (www.prisma-statement.org). A clinical librarian was consulted for assembling of the systematic search strategy for multiple databases, including PubMed, EMBASE and Web of Science, to identify all relevant publications. The search strategy included (MeSH) terms and free text related to or describing CD, perianal disease and ‘red flags/diagnosis’. The complete search strategy is presented in Supplementary Table 1. The final search was performed on 3 July 2023. A search filter for human studies was used. There were no publication date restrictions. Reference lists and bibliographies of included studies were hand searched for relevant studies and trial and study registries were searched for relevant ongoing studies. Abstracts were screened independently by two reviewers (L.M. and G.M.). Data were extracted from the selected studies in duplicate by two reviewers (L.M. and G.M.). Any disagreements were consulted with the senior investigators (J.B., C.B.). A detailed protocol was registered in the PROSPERO database prior to this research (registration no.: CRD42022365616).

#### Inclusion and exclusion criteria

Studies were included if they met the following criteria: (1) patients presenting with a PAA and/or PAF, (2) previously unknown CD, (3) reporting on clinical characteristics associated with CD diagnosis, (4) including patients ≥ 16 years, (5) peer reviewed and (6) minimum of ten patients included in the studies. Studies were excluded if they were: (1) presenting results of fistulas with other well-known aetiology (iatrogenic, malignancy, pilonidal sinus), (2) reviews and case reports, (3) animal studies and (4) publications in languages other than English or Dutch.

#### Outcomes

Study characteristics (year of publication, country of publication, number of participating centres, study design, study period and number of included patients), demographic data (patients characteristics, age and sex) and outcome data were recorded. The primary outcome was identification of clinical characteristics associated with CD in patients presenting with a PAA or PAF. In addition, the reported incidence and the median time to diagnosis of CD in patients with perianal disease from the selected studies was assessed. Quality assessment of all included studies was performed by use of the Newcastle–Ottawa scale (NOS, see Supplementary Table 2) [37].

### Part II: retrospective matched cohort study

#### Inclusion and exclusion criteria

All adult patients (≥ 16 years) presenting with a first or recurrent PAF and eventually diagnosed with CD at the Flevoziekenhuis (a non-academic teaching hospital in the Netherlands with dedicated inflammatory bowel disease [IBD] care) between September 2012 and March 2023 were identified from a prospective administrative database. Patients with a CD diagnosis prior to presentation with a PAF were excluded. CD Patients were matched 1:3 to patients with a cryptoglandular PAF from the same study period (presenting in the same month with a PAF), who served as controls.

This study was approved by the accredited Medical Ethics Committee (METC) (reference number W21_281#21.308) and conducted according to the principles of the Declaration of Helsinki (64th WMA General Assembly, Fortaleza, Brazil, October 2013). All patients were informed in writing and were offered to withdraw from participation (opt-out).

#### Data collection

Between September and December 2023, a questionnaire with items identified in the systematic review, supplemented with items from the IO-IBD red flags index for luminal CD, was administered by the investigator to all included patients. Patients were asked to recall symptoms and signs experienced during their first PAF.

#### Statistical analyses

The proportion of patients reporting the selected items in patients presenting with Crohn’s PAF were compared with those reported in patients with a cryptoglandular PAF. Continuous data were presented as means and standard deviation (SD) or median and interquartile range (IQR) according to the distribution of the data. Categorical data were presented as number of patients and proportions. Univariate analyses were performed to identify items associated with CD diagnosis. All items with a *p*-value < 0.25 in this analysis were included in a multivariate logistic regression analysis. A backward selection was performed in the logistic regression on the basis of the *p*-value of the Wald statistic. A two-tailed *p*-value of less than 0.05 was considered significant and a receiver operating characteristic (ROC) analysis was conducted to determine the threshold that discriminated CD from non-CD fistulas. All analyses were performed by use of SPSS Statistics for Windows (version 22, IBM Crop., Armonk, New York, USA).

## Results

### Part I: systematic review

**Fig. 1:**
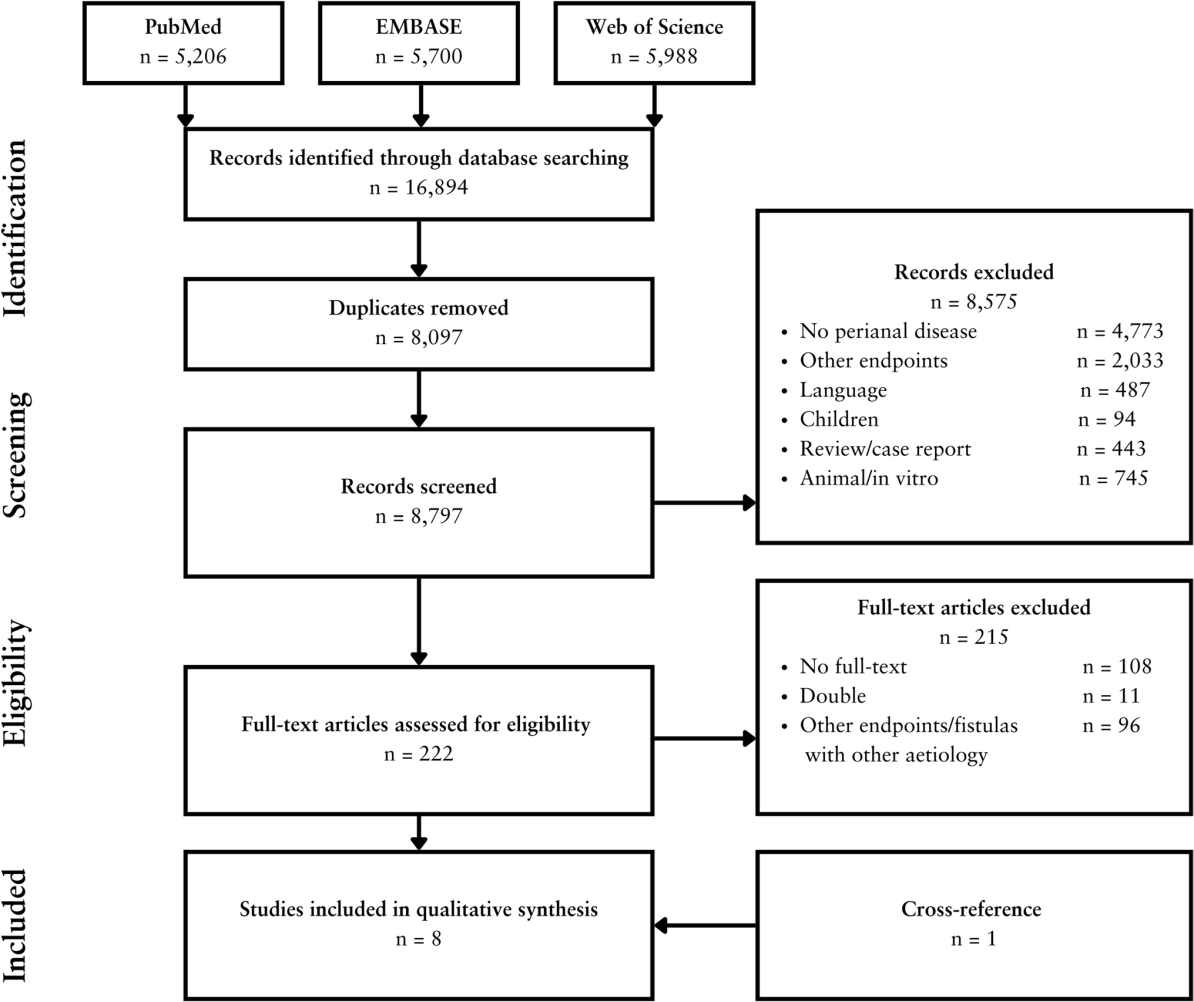
Flowchart of the search and selection procedure of studies

The PRISMA flow diagram of the search and selection process is presented in Fig. 1. From a total of 16,894 references, 8 articles were included comprising 5 retrospective cohort studies [36, 37, 40–42], 2 prospective cohort studies [38, 39] and 1 case–control study [12], resulting in a total number of 22,481 patients. Study quality ranged from moderate (some risk of bias, *n* = 4) to good (low risk of bias, *n* = 4). Total scores of the quality assessment according to the NOS are summarised in supplementary tables 3 and 4.

#### Signs and symptoms associated with CD

Study characteristics of all included studies are summarised in Supplementary Table 5. A summary of all findings, including patient demographics, clinical characteristics and symptoms, is presented in Table 1.

**Table 1:** Summary of findings

Study	Sex (male, *n*, %)	Age (years)	Smoking (*n*, %)	BMI (mean, SD)	Family history of CD (*n*, %)	Abdominal pain (*n*, %)	Diarrhoea (*n*, %)	Fever (*n*, %)	Weight loss (*n*, %)	Anaemia (*n*, %)	Rectal blood loss (*n*, %)	Extra-intestinal manifestations	Diabetes	Perianal surgery history (*n*, %)	Previous abdominal surgery (*n*, %)
Xu et al. [38]	107 (77)	28.2*	NR	NR	NR	30 (23)	64 (46)	27 (19.4)	37 (26.6)	21 (15.1)	23 (16.5)	NR	NR	90 (64.7, with 23% ≥ 1 surgery)	NR
George et al. [39]	Non-CD: NR CD: 10 (63)	All: NR CD: 28.0 (IQR NR, all CD patients < 40 years)	NR	NR	NR	NR	NR	NR	NR	NR	NR	NR	NR	NR	NR
Haddow et al. [40]	All: 46 (75) Non-CD: 38 (79) CD: 8 (62)	Non-CD: 41.1* (11.6) CD: 36.1* (18.5)	NR	NR	NR	NR	NR	NR	NR	NR	NR	NR	NR	Non-CD: 1 (IQR 0–4) CD: 0 (IQR 0–4)	Non-CD: 0 (0) CD: 6 (46)
Coremans et al. [41]	All: 110 (60) Non-CD: 87 (73.1) CD: 23 (36.5)	Non-CD: 48.9* (± 1.4) CD: 38.5* (± 1.8) *p* = 0.0001	NR	NR	NR	NR	NR	NR	NR	NR	NR	NR	NR	NR	NR
Yzet et al. [42]	66 (71) Univariate¶: 0.51 (0.11–2.44, *p* = 0.396) Multivariate¶: NR	43 (IQR 34–56) Univariate¶: 0.9 (0.9–1.0, *p* = 0.043) Multivariate¶: 1.5 (0.1–2.0, *p* = 0.140)	All: 23 (25) Univariate¶: 0.4 (0.1–1.9, *p* = 0.26) Multivariate: NR	NR	All: 2 (2) Univariate¶: 1.7 (1.0–2.3, *p* = 0.02) Multivariate¶: 1.5 (0.1–2.5, *p* = 0.148)	All: 2 (2) Univariate¶: 1.7 (1.0–2.6, *p* = 0.020) Multivariate¶: 3.2 (1.2–3.7, *p* = 0.002)	All: 2 (2) Univariate¶: 5.8 (2.9–7.3, *p* < 0.001) Multivariate¶: 5.2 (2.5–7.1, *p* < 0.001)	NR	All: 4 (4) Univariate¶: 6.2 (3.5–10.9, *p* < 0.001) Multivariate¶: 4.3 (2.4–7.7, *p* < 0.001)	NR	NR	All: 4 (4) Univariate¶: 1.2 (0.2–2.4, *p* = 0.18) Multivariate¶: 1.1 (0.1–2.9, *p* = 0.292)	NR	All: 10 (11) Univariate¶: 0.9 (0.3–2.8, *p* = 0.35) Multivariate¶: NR	NR
Oliveira et al. [43]	All: 83 (66) Non-CD: 18 (60) CD: 65 (67.7)	Non-CD: 42.4* (± 14.7) CD: 28.6* (± 14.9) *p* < 0.0001	NR	NR	NR	NR	NR	NR	NR	NR	NR	NR	NR	NR	NR
Thomas et al. [17]	All: 29,208 (65) PAA cases: 11,875 (66.4) Controls: 17,333 (63.9)	PAA group: 45.8* (15.6) Controls: 46.9* (15.2) 20 < × ≤ 40 years¶: 0.8 (0.4–1.6, *p* = 0.52) 40 < × ≤ 60 years¶: 0.3 (0.1–0.7. *p* = 0.005) 60 < × ≤ 80 years¶: 0.4 (0.2–0.9, *p* = 0.04) ≥ 80 years¶: 0.1 (0.0–1.2, *p* = 0.07)	Never smoker¶: 1 (NR) Current smoker¶: 0.9 (0.6–1.3, *p* = 0.59) Ex-smoker¶: 1.2 (0.7–1.9, *p* = 0.58)	PAA cases: 28.1* (6.2) Controls: 27.2* (4.9) *p* < 0.0001 < 25¶: 1 (NR) 25–30¶: 0.82 (0.52–1.28, *p* = 0.38) > 30¶: 0.43 (0.24–0.78, *p* = 0.005)	NR	PAA cases with CD: 43 (30.7) ¶: 1.33 (0.91–1.93, *p* = 0.14)	PAA cases: 60 (0.34, prior to PAA) PAA cases with CD: 40 (28.6) ¶: 1.3 (0.9–2.0, *p* = 0.18) Use of anti-diarrhoeals¶: 2.7 (1.7–4.3, *p* < 0.0001)	NR	NR	PAA cases: 1121 (6.3) Controls: 1384 (5.1) *p* < 0.0001 Men¶: 2.8 (1.3–5.9, *p* = 0.002) Women¶: 1.2 (0.5–2.9, *p* = 0.63)	PAA cases with CD: 19 (13.6) ¶: 1.6 (1.0–2.6, *p* = 0.05)	PAA cases: 0 (before diagnosis of IBD)	PAA cases: 1700 (9.5) Controls: 1619 (6) *p* < 0.0001 ¶: 0.4 (0.2–1.2, *p* = 0.1)	NR	NR
Hokkanen et al. [44]	All: 2106 (62.9) Non-CD: 1538 (59.9) CD: 568 (72.8)	Non-CD, *n* (%): 0–19 years: 127 (4.9) 20–39 years: 862 (33.6) 40–59 years: 1091 (42.5) 60–79 years: 441 (17.2) 80+ years: 74 (2.9) CD, *n* (%): 20–39 y: 365 (46.8) 40–59 years: 288 (36.9) 60–79y: 84 (10.8)	NR	NR	NR	NR	NR	NR	NR	NR	NR	Hidradenitis: Non-CD (prevalence/100): 2017: 1.6 CD (prevalence/100): 2017: 4.2	Non-CD (prevalence/100) 2017: 8.9 CD (prevalence/100): 2017: 4.9	NR	NR

Study	Fistula type (*n*, %)	Internal fistula openings (*n*, %)	External fistula openings (*n*, %)	Other perianal diseases (*n*, %)	Rectal involvement (*n*, %)	Delay (months)
Xu et al. [38]	Simple: 13 (9.4) Complex: 126 (90.6)	NR	1: 48 (34.5) 2: 44 (31.7) ≥ 3: 47 (33.8)	Fissure: 6 (4.3) Abscess: 33 (23.7) Rectovaginal fistula: 7 (5) Verrucous skin 43 (30.9)	NR	NR
George et al. [39]	NR	NR	NR	NR	NR	13 patients (81%): CD diagnosis within 36 months of PAA
Haddow et al. [40]	Secondary tracts/supralevator: Non-CD: 3 (6) CD: 4 (31), *p* = 0.02	NR	NR	Collections: Non-CD: 16 (33) CD: 12 (92), *p* < 0.001	Non-CD: 1 (2) CD: 7 (54), *p* < 0.001	NR
Coremans et al. [41]	Curved or radial	IO anterior to TAL: Non-CD: 39 (32.8) CD: 47 (74.6) *p* = 0.017 IO posterior to TAL: Non-CD: 74 (62.2) CD: 31 (49.2) *p* = 0.024	EO anterior to TAL: Non-CD: 45 (37.2) CD: 37 (52.8) *p* = 0.035 EO posterior to TAL: Non-CD: 73 (60.3) CD: 29 (41.4) *p* = 0.012	NR	NR	NR
Yzet et al. [42]	Simple (all): 19 (20), univariate¶: 0.85 (0.15–4.68, *p* = 0.913) Complex (all): 2 (2), univariate¶: 0.4 (0.1–2.8, *p* = 0.686)	NR	NR	Haemorrhoids (all): 15 (16), univariate¶: 1.2 (0.1–10.3, *p* = 0.90) Fissure (all): 4 (4), univariate¶: 0.6 (0.4–3.8, *p* = 0.57) Abscess (all): 48 (52), univariate¶: 1.0 (0.2–4.9, *p* = 0.97)	NR	7.6 (2.7–26.1)
Oliveira et al. [43]	Transsphincteric: Non-CD: 15 (50) CD: 37 (38.5) Intersfincteric: Non-CD: 10 (33.3) CD: 34 (35.42) Suprasphincteric: Non-CD: 0 (0) CD: 1 (1.04) Multiple branched: Non-CD: 5 (16.7) CD: 24 (25), *p* = 0.34	NR	NR	Abscess: Non-CD: 9 (30) CD: 32 (33.3) *p* = 0.73	Non-CD: 2 (6.7) CD: 29 (30.2) *p* = 0.0009	NR
Thomas et al. [17]	NR	NR	NR	NR	NR	20.64 (11.16–44.4)
Hokkanen et al. [44]	NR	NR	NR	NR	Non-CD (prevalence/100): 2017: 1.8 CD (prevalence/100): 2017: 4.6	NR

*CD* Crohn’s disease, *PAA* perianal abscesses, *PAF* perianal fistula, *BMI* body mass index, *NR* not reported, *IQR* interquartile range, *p* *p*-value/significance, *QoL* quality of life, *IO* internal fistula opening, *EO* external fistula opening, *TAL* transverse anal line

*Mean (SD)

¶ Hazard ratio (95% confidence interval)

Age and sex were described in all studies [17, 38–43]. Patients with CD were younger at presentation with a PAF compared with patients without CD diagnosis (overall mean from three studies presenting data 34.4 versus 44.1 years respectively *p* < 0.0001) [40, 41, 43]. Although Hokkanen et al. identified male as a risk factor for PAF [44], no study identified sex as a risk factor for CD. Smoking was described in one study without a correlation with future CD diagnosis in current smokers (HR 0.9 [95% CI 0.6–1.3], *p* = 0.59) or ex-smokers (HR 1.2 [95% CI 0.7–1.9], *p* = 0.58) [17]. Diabetes was described in two studies [17, 44], but despite a significantly higher prevalence of diabetes in the PAA group compared with healthy controls (9.5% versus 6% respectively, *p* = 0.0001), it was no significant predictor for underlying CD [17]. In one study an association was found between a positive family history of IBD and the development of CD in univariate analysis in patients undergoing surgery for a PAF (HR 1.7 [95% CI 1.0–2.3], *p* = 0.021) [42].

Abdominal pain and diarrhoea as clinical symptoms in patients with PAA/PAF eventually diagnosed with CD were frequently described (23% and 30.7%, respectively, and 46% and 28.6%, respectively) [17, 38, 42]. One study showed a significant association between abdominal pain and development of CD in univariate and multivariate analysis (HR 1.7 [95% CI 1.0–2.6], *p* = 0.020 and HR 3.2 [95% CI 1.2–3.7], *p* = 0.020) [42], whereas diarrhoea was found to be related in two out of three studies [17, 38, 42]. Cox regression analysis showed that the use of medication for diarrhoea within 180 days of PAA diagnosis was associated with an increased likelihood of a future CD diagnosis (HR 2.7 [95% CI 1.7–4.3, *p* < 0.0001) [17]. In the study by Yzet et al., an association between chronic diarrhoea and the diagnosis of CD was found in univariate analysis (HR 5.8 [95% CI 2.9–7.3], *p* < 0.001) as well as in multivariate analysis (HR 5.2 [95% CI 2.5–7.1], *p* < 0.001) [42]. Fever as a clinical symptom in patients with perianal fistulising CD was only analysed in one study, but was not found to have any prognostic significance [38]. Weight loss, anaemia and rectal blood loss were found in 26.6%, 15.1% and 16.5%, respectively, of all patients with perianal fistulising CD [38]. Yzet et al. demonstrated an association between weight loss and the development of CD in univariate analysis (HR 6.2 [95% CI 3.5–10.9], *p* < 0.001) and multivariate analysis (HR 4.3 [95% CI 2.4–7.7], *p* < 0.001) [42], and Thomas et al. showed that anaemia in men (HR 2.8 [95% CI 1.3–5.9], *p* = 0.002), but not in women, and rectal blood loss (HR 1.6, 95% CI 1.0–2.6, *p* = 0.05) were associated with an increased likelihood of CD diagnosis in the future [17]. Extra intestinal manifestations were described in three studies [17, 42, 44], but no association with the diagnosis of CD could be demonstrated [42].

Previous abdominal and perianal surgery, described in three studies, were frequently seen before the diagnosis of CD (up to 64.7%, with 23% having ≥ 1 perianal surgery) [38], but only previous abdominal surgeries were more frequently seen in the CD cohort (46%, versus 0 patients in the non-CD cohort, *p*-value not reported) [40].

Complex PAF were found more frequently in CD patients than simple PAF (90.6% versus 9.4%, respectively) [38]. In addition, multiple fistula tracts were observed significantly more often in patients with CD as compared with non-CD patients (31% versus 6%, *p* = 0.02) [40], with 31.7% having two external fistula openings and 33.8% having ≥ 3 external fistula openings. Moreover, in patients with CD, a PAF with an anterior internal opening was more frequently seen (74.6% versus 32.8%, *p* = 0.0169). Rectal involvement was mentioned in two studies, showing that patients with CD significantly had rectal involvement more often as compared with non-CD patients (54% versus 2%, *p* < 0.001 [40] and 30.2% versus 6.7%, *p* = 0.0009 [43], respectively).

#### Incidence of CD and delay in diagnosis

A total of three studies reported on the incidence and time to diagnosis of CD in patients with a PAA (*n* = 2) [17, 39] and/or PAF (*n* = 1) [42]. The incidence of CD was significantly lower in patients presenting with PAA than PAF (0.8%, 156/18.532 versus 7.5%, 7/93, respectively, *p* < 0.0001) [17, 39, 42]. Median reported time to diagnosis in the selected studies ranged from 7.6 (IQR 2.7–26.1) to 20.6 (IQR 11.2–44.4) months with a mean follow-up ranging from 36.0 to 67.2 months [17, 39, 42].

### Part II: retrospective matched cohort study

A total of 25 patients with PAF, eventually diagnosed with CD, and 75 matched patients with cryptoglandular PAF, were included. All patients returned the questionnaires (100% response rate). Median follow-up in all patients was 5 years (IQR 2–10).

**Fig. 2:**
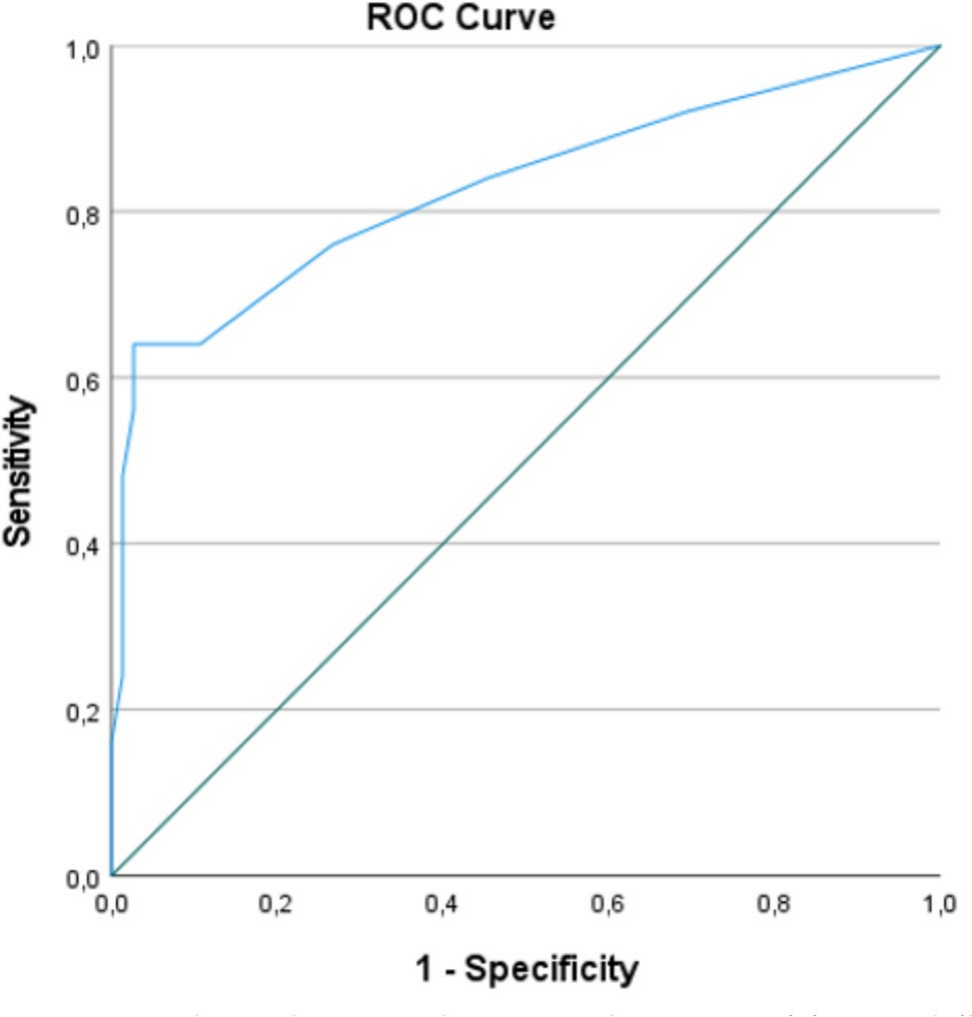
Receiver operating curve analysis showing that a combination of four red flags (age < 40 years,  > 2 previous perianal interventions, weight loss and abdominal pain) was associated with good discrimination of CD versus non-CD (area under the curve [AUC] 0.828, 95% CI 0.719–0.937)

**Fig. 3:**
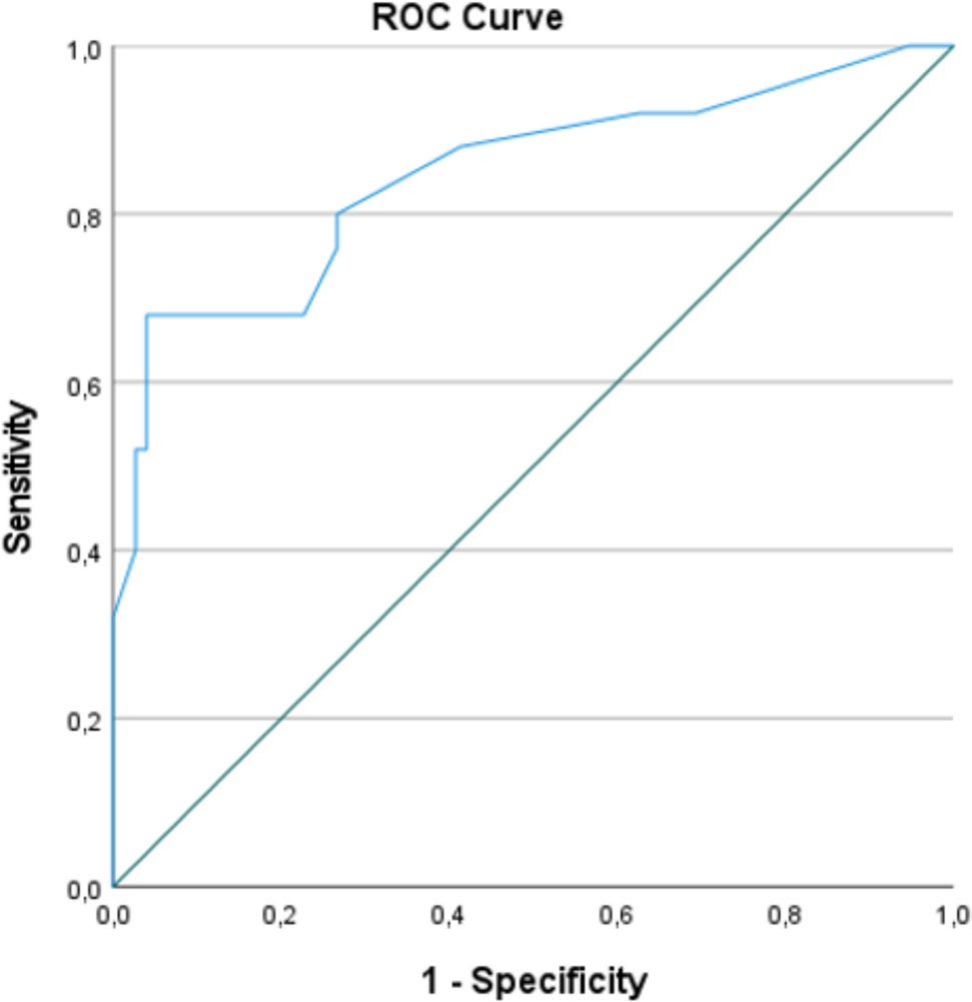
Adding the additional items associated with CD in univariate analysis (age at first PAF, a positive family history of IBD, a higher number of previous perianal surgical interventions, weight loss [> 5% in 3 months], abdominal pain diarrhoea and fatigue) only marginally improved the discriminative value (AUC 0.845, 95% CI 0.745–0.946)

Table 2 presents the baseline characteristics and the proportion of patients with a positive answer on the questionnaire for patients in the CD group compared with patients in the cryptoglandular group. A total of 7 out of 28 items were significantly more frequent in the CD group compared with the cryptoglandular group: age at first PAF, a positive family history of IBD, a higher number of previous perianal surgical interventions, weight loss (> 5% in 3 months), abdominal pain, diarrhoea and fatigue. Age and number of previous perianal interventions were assessed as a continuous and categorical variable (< 40 years old or ≥ 40 years old and ≤ 2 or > 2 perianal interventions), with both categorical variables being significantly more associated with the outcome measure of interest. Therefore, those items were included as a categorical variable in the multivariate analysis. In total, four independent items were found to be significantly related to a diagnosis of CD, which were included in a red flags index: age < 40 years (OR 3.4, 95% CI 1.0–11.5, *p* = 0.042), > 2 previous perianal interventions (OR 3.4 [95% CI 1.0–10.1], *p* = 0.050), weight loss (> 5% in 3 months, OR 14.4 [95% CI 3.7–55.6], *p* < 0.001) and abdominal pain (OR 9.8 [95% CI 1.9–49.8], *p* = 0.006, Table 3), with a receiver operating curve (ROC) analysis showing that a combination of these red flags was associated with good discrimination of CD versus non-CD (area under the curve, AUC, 0.83 [95% CI 0.72–0.94], Fig. 2). Adding the additional items associated with CD in univariate analysis only marginally improved the discriminative value (AUC 0.85 [95% CI 0.75–0.95], Fig. 3).

**Table 2:** Associations between a positive answer in the pre-determined questionnaire in cryptoglandular as well as patients with CD

Red flags	CD *n* = 25	Cryptoglandular *n* = 75	OR (95% CI)	*p*-value
Gender (women, *n*, %)^a^	12 (48)	26 (35)	1.7 (0.7–4.4)	0.234
Age first PAA/PAF (median, IQR)^a^	28 (21–43)	41 (32–50)	NA	< 0.001*
BMI (median, IQR)^a^	25 (24–29)	27 (24–30)	NA	0.299
Smoking (*n*, %)^a,b^	8 (32)	22 (29)	1.1 (0.4–3.0)	0.801
Family history (*n*, %)^a,b^	5 (20)	4 (5)	4.4 (1.1–18.1)	0.038*
Number of previous perianal interventions (median, IQR)^a^	4 (2–7)	2 (0–3)	NA	0.002*
Bowel surgery in the past (*n*, %)^a^	3 (12)	4 (5)	2.4 (0.5–11.7)	0.362
Weight loss (> 5% in 3 months, *n*, %)^a,b^	12 (48)	5 (7)	12.9 (3.9–42.9)	< 0.001*
Abdominal pains (*n*, %)^a,b^	8 (32)	3 (4)	11.3 (2.7–47.1)	< 0.001*
Abdominal pain childhood (*n*, %)^a^	6 (24)	7 (9)	3.1 (0.9–10.2)	0.084
Diarrhoea (*n*, %)^a,b^	8 (32)	3 (4)	11.3 (2.7–47.1)	< 0.001*
Rectal bleeding (*n*, %)^a,b^	11 (44)	24 (32)	1.7 (0.7–4.2)	0.276
Fatigue (*n*, %)^a^	13 (52)	18 (24)	3.4 (1.3–8.8)	0.009*
Anaemia (*n*, %)^a,b^	4 (16)	14 (19)	0.8 (0.2–2.8)	1.000
Vitamin deficiencies (*n*, %)^a^	10 (40)	19 (25)	1.9 (0.8–5.1)	0.162
IBS (*n*, %)^b^	2 (8)	9 (12)	0.6 (0.1–3.1)	0.726
Ulcerative colitis (*n*, %)^b^	1 (4)	0 (0)	NA	0.250
Coeliac disease (*n*, %)^b^	0 (0)	2 (3)	NA	1.000
Lactose intolerance (*n*, %)^b^	3 (12)	6 (8)	1.6 (0.4–6.8)	0.687
Rheumatoid arthritis (*n*, %)^b^	6 (24)	8 (11)	2.6 (0.8–8.6)	0.108
Ankylosing spondylitis (*n*, %)^b^	2 (8)	0 (0)	NA	0.061
Alopecia (*n*, %)^b^	3 (12)	6 (8)	1.6 (0.4–6.8)	0.687
Psoriasis (*n*, %)^b^	3 (12)	7 (9)	1.3 (0.3–5.6)	0.708
Erythema nodosum (*n*, %)^b^	3 (12)	3 (4)	3.3 (0.6–17.4)	0.163
Skin diseases (*n*, %)^b^	3 (12)	3 (4)	3.2 (0.6–17.4)	0.163
Hidradenitis (*n*, %)^b^	4 (16)	3 (4)	4.6 (0.9–22.1)	0.063
Uveitis (*n*, %)^b^	6 (24)	6 (8)	3.6 (0.9–12.6)	0.068
Stomatitis aphtosis (*n*, %)^b^	6 (24)	11 (15)	1.8 (0.6–5.6)	0.357

*n* number, *IQR* interquartile range

^a^Derived from literature search

^b^Derived from IO-IBD Red Flag Index

*Significant *p*-value

**Table 3:** Items independently associated with CD diagnosis after multivariate logistic regression analysis with backward selection using the Wald statistic

Red flags	OR (95% CI)	*p*-value	Rounded coefficient
Age < 40 years	3.4 (1.0–11.5)	0.042	3
> 2 previous perianal interventions	3.4 (1.0–10.1)	0.050	3
Weight loss (> 5% in 3 months)	14.4 (3.7–55.6)	< 0.001	14
Abdominal pains	9.8 (1.9–49.8)	0.006	10

## Discussion

Literature on clinical characteristics (red flags) of CD in patients presenting with a PAA and/or PAF is scarce. The current systematic review demonstrated that the incidence of CD in patients with only a PAA was negligible, whereas this was 7.5% in patients presenting with PAF. The matched controlled retrospective study identified four items that were independently associated with underlying CD. Patients with CD were significantly younger and had more weight loss, abdominal pain and previous perianal interventions. Collecting these items in a perianal red flags index yielded a good discriminative value of CD versus non-CD (AUC 0.83 [95% CI 0.72–0.94]).

PAFs are the first manifestation in up to 10% of all patients with Crohn’s [45], and identifying specific patient characteristics in this population can be challenging [12, 46]. A population-based study showed that one-third of patients with CD had perianal surgery in the 5 years preceding their diagnosis [13]. The systematic review also showed a reported median time from perianal disease to CD diagnosis ranging from 7.6 (IQR 2.7–26.1) to 20.6 (IQR 11.2–44.4) months [17, 39, 42], which is longer then the time to diagnose CD in patients presenting with abdominal complaints [17–20, 30, 42]. This emphasises the need to improve early diagnosis, since literature shows that patients with perianal CD generally have a more aggressive disease phenotype [47] with increased risk of undergoing major surgeries during the course of their disease when compared with patients without perianal CD (e.g. a colonic resection and/or stoma) [48], and without timely multidisciplinary management, a late diagnosis will put patients at higher risk of unnecessary damage to the sphincter, progression of the disease and decreased quality of life (QoL) [20–22].

Early recognition starts with the identification of signs and symptoms suggestive of CD in patients presenting with perianal disease [17–20, 42]. Using a perianal red flags index with only four items could be an easy tool for all (non-IBD) doctors. Previously, the International Organization for the Study of Inflammatory Bowel Diseases (IO-IBD) identified red flags for luminal CD in patients presenting at a general practitioners’ service and a high red flag index ≥ 8 was shown to discriminate CD from irritable bowel syndrome (IBS) and healthy subjects (OR 205, *p* < 0.0001) [31]. Non-healing or complex PAA or PAF or perianal lesions had the highest odds ratio (OR) (> 50) for suspicion of CD, however, the discriminating value of red flags in patients presenting with a PAA or PAF as a first symptom was not investigated.

Given the long delay in diagnosis from the reported studies, it may be concluded that diagnosing CD on clinical grounds alone seems suboptimal. Although we should be alert to CD signs and symptoms, possible non-invasive biomarkers (e.g. fecal calprotectin [FCP] or other biomarkers) should be considered [49]. FCP has been demonstrated to be able to differentiate IBD from IBS in the general practitioner’s practice [50, 51], and Stevens et al. recently showed that a FCP measurement of ≥ 150 mcg/g discriminates perianal CD from cryptoglandular PAF, even in the absence of intestinal ulcers [52]. In addition, magnetic resonance imaging (MRI) and/or ultrasound (US) should be considered in patients suspected of having CD [53] to identify patients at high risk of having CD early.

To our knowledge, this is the first study that systematically analysed signs and symptoms associated with CD in patients presenting with a PAF and it is the first study that systematically asked matched patients to recall the CD related symptoms experienced during their first PAF. Moreover, since patients were retrospectively included from 2012, a long period of follow-up was available, which makes a missed diagnosis of CD unlikely. Secondly, this study was conducted at a non-academic centre, which makes results generalisable and representative of the average patient (CD or non-CD) presenting with perianal disease.

This study also has several limitations. The number of patients was limited and as a result of the retrospective design of this study potential bias was unavoidable, including information gathering bias and selection bias (e.g. absence of medical records or potential lacking of information in medical records, which may have led to missing data and heterogeneity of the observed medical records). This was attempted to be solved by adding a matched cohort of patients with cryptoglandular PAF to identify all clinically relevant confounders. However, recall bias due to a long median follow-up was unavoidable. Moreover, since patients were retrospectively asked to recall symptoms and signs experienced during their first PAF, it was not possible to collect specific data on fistula characteristics (e.g. complexity). It is assumed that the perianal red flags index can be improved by incorporating fistula characteristics and/or biomarkers (e.g. FCP) as well. Obviously, findings will need to be confirmed in larger, prospective studies.

In conclusion, this study identified several red flags for CD in patients presenting with a PAF. Using these items as a simple and feasible ‘perianal red flags index’, a reliable discrimination can be made between patients at risk for CD and non-CD. The tool is intended to help clinicians in primary or secondary care, and potentially patients, to consistently identify symptoms and signs that might lead to the diagnosis of CD at an early stage. Hopefully, this perianal red flags index will reduce the time to CD diagnosis, eventually enabling intervention at a an early stage to alter disease progression.

## Supplementary Information

Below is the link to the electronic supplementary material.

Supplementary file1 (DOCX 38 KB)

## Data Availability

The authors of this manuscript confirm that the data supporting the findings of this manuscript are available within this manuscript or in its supplementary materials.
