# Supplementary material for: Early diagnosis of Crohn’s disease in patients presenting with a perianal fistula: systematic review and development of a perianal red flags index
Source: Tech Coloproctol. 2025 Mar 28;29(1):89. doi: 10.1007/s10151-024-03106-y (PMC11953225; doi:10.1007/s10151-024-03106-y)
Supplement: Supplementary file 1 — Supplementary file1 (DOCX 38 KB) [file 10151_2024_3106_MOESM1_ESM.docx]

**SUPPLEMENTARY TABLES**

| Database | Art. found 3 Jan 2022 | Art. retrieved after subtrac­tion | Unique articles | Art. found 11 Jul 2022 | Art. retrieved after dedupli­cation | Art. found 3 Jul 2023 | Art. retrieved after dedupli­cation |
| --- | --- | --- | --- | --- | --- | --- | --- |
| Medline | 4833 | 4833 | 4826 | 5041 | 199 | 5206 | 126 |
| Embase (no conference publications) | 5244 | 1707 | 1371 | 5470 | 57 | 5700 | 130 |
| Web of Science | 5440 | 2155 | 1895 | 5763 | 113 | 5988 | 80 |
| Total |  |  | 8092 | 16274 | 369 | 16894 | 336 |

**Supplementary table 1** Complete search strategy

|  | Case-control studies | Cohort studies |
| --- | --- | --- |
| Selection  4 stars | 1. Is the case definition adequate?  2. Representativeness of the cases  3.Selection of controls 4. Definition of controls | 1. Representativeness of the exposed cohort 2. Selection of the non-exposed cohort 3. Ascertainment of exposure 4. Demonstration that outcome of interest was not present at start of study |
| Comparability  2 stars | 1. Comparability of cases and controls on the basis of the design or analysis | 1. Comparability of cohorts at the basis of the design or analysis |
| Exposure/outcome  3 stars | 1. Ascertainment of exposure 2. Same method of ascertainment for cases and controls 3. Non-response rate | 1. Assessment of outcome 2. Was follow-up long enough for outcomes to occur 3. Adequacy of follow up of cohorts |

**Supplementary table 2** Key-elements used for assessing risk of bias of cohort studies and case series according to the Newcastle Ottawa Scale (NOS) [37]

|  | Selection | | | | Comparability | | Exposure | | | Total  (9/9) |
| --- | --- | --- | --- | --- | --- | --- | --- | --- | --- | --- |
| **Study** | Adequate definition of the case | Representativeness of the case | Selection of controls | Definition of controls | Main factor | Additional factor | Ascertainment of exposure | Same method of ascertainment for cases and controls | Non-response rate |  |
| Thomas et. al [12] | X | X | X | X | X | X | X | X | X | 9/9 |

**Supplementary table 3** Newcastle-Ottawa quality assessment scale for all included case-control studies (n= 1), X = one star

|  | Selection | | | | | Comparability | | Outcome | | | Total (9/9) |
| --- | --- | --- | --- | --- | --- | --- | --- | --- | --- | --- | --- |
| **Study** | Representativeness of the exposed cohort | Selection of the non-exposed cohort | Ascertainment of exposure | Outcome of interest not present at the start of the study | | Main factor | Additional factor | Assessment of outcomes | Sufficient follow-up time | Adequacy of follow-up |  |
| Xu et. al [36] | X | 0 | X | | 0 | 0 | 0 | X | X | X | 5/9 |
| George et. al [37] | X | 0 | X | | X | X | 0 | X | X | X | 7/9 |
| Haddow et. al [38] | X | X | X | | X | X | 0 | X | 0 | 0 | 6/9 |
| Coremans et. al [39] | X | X | X | | 0 | X | 0 | X | 0 | 0 | 5/9 |
| Yzet et. al [40] | X | X | X | | X | X | X | X | X | X | 9/9 |
| Oliveira et. al [41] | X | X | X | | X | X | 0 | X | 0 | 0 | 6/9 |
| Hokkanen et. al [42] | X | X | X | | 0 | X | X | X | X | X | 8/9 |

**Supplementary table 4** Newcastle-Ottawa quality assessment scale for all included cohort studies (n= 7), X = one star

| Author | Year of publication | Country | Centres | Study design | Study period | Patients (n) | Aim | Cohort of patients | CD patients (total) |
| --- | --- | --- | --- | --- | --- | --- | --- | --- | --- |
| Xu et. al [38] | 2019 | China | Single | Retrospective cohort | 2010-2017 | 139 | To explore the clinical features of anal fistula in CD | Perianal fistulising CD patients | 139 (139) |
| George et. al [39] | 2018 | UK | Single | Retrospective cohort | 2004-2016 | All: 678  Non-CD: 662  CD: 16 | To identify details of all patients with PAA (and those eventually developing CD) | All patients with PAA | 16 (678) |
| Haddow et. al [40] | 2019 | UK | Single | Prospective cohort | 2014-2015 | All: 61  Non-CD: 48  CD: 13 | To compare clinical phenotypes of idiopathic and CD-related PAF | Patients undergoing surgery for PAF | 13 (61) |
| Coremans et. al [41] | 2003 | Belgium | Single | Prospective cohort | 2000-2002 | All: 182  Non-CD: 119  CD: 63 | Comparing characteristics of anal fistulas in male/female CD and non-CD patients | Patients with PAF | 63 (182) |
| Yzet et. al [42] | 2020 | France | Single | Retrospective cohort | 2008-2017 | 93 | To evaluate the risk of CD in patients undergoing PAF surgery | All patients undergoing surgery for PAF | 7 (93) |
| Oliveira et. al [43] | 2016 | USA | Single | Retrospective cohort | 2012-2015 | All: 126  Non-CD: 30  CD: 96 | To evaluate MRI imaging differences of PAF in patients with and without CD | Patients with PAF + MRI | 96 (126) |
| Thomas et. al [17] | 2019 | UK | Population based | Case-control | 1995-2017 | All: 44965  PAA cases: 17854  Controls: 27111 | To assess the future risk of IBD/to identify potential predictors for IBD after PAA | Patients with PAA matched to up to two controls without PAA | PAA cases: 140 (17854)  Matched controls: 25 (27111) |
| Hokkanen et. al [44] | 2019 | UK | Population based | Retrospective cohort | 2014-2017 | Non-CD: 2568  CD: 780 | To generate population-based real-world evidence on the epidemiology of PAF | All subjects registered in a primary care general practice with diagnosis of PAF | 780 (3348) |

**Supplementary table 5** Study characteristics of all included studies (CD = Crohn’s disease, PAA = perianal abscesses, PAF = perianal fistula, IBD = Inflammatory Bowel Disease**,** MRI = Magnetic Resonance Imaging)
